# Complexity of plantar flexor torque and gait covaries in older adults: a network physiology approach to neuromuscular aging

**DOI:** 10.3389/fnetp.2026.1947848

**Published:** 2026-10-07

**Authors:** Philipp Bauer, João S. Gomes, Tiago Marques, Sandrina Lourenço, João H. Oliveira, Jamie Tallent, Nelson Cortes, João R. Vaz

**Affiliations:** 1 Egas Moniz Center for Interdisciplinary Research (CiiEM), Egas Moniz School of Health & Science, Caparica, Portugal; 2 Neuromuscular Research Laboratory, Faculty of Human Kinetics, University of Lisbon, Lisbon, Portugal; 3 CIPER, Faculty of Human Kinetics, University of Lisbon, Lisbon, Portugal; 4 School of Sport, Rehabilitation and Exercise Sciences, University of Essex, Colchester, United Kingdom; 5 Monash Exercise Neuroplasticity Research Unit, Department of Physiotherapy, School of Primary and Allied Healthcare, Faculty of Medicine, Nursing and Health Science, Monash University, Melbourne, VIC, Australia; 6 Department of Bioengineering, College of Engineering and Computing, George Mason University, Fairfax, VA, United States

**Keywords:** fall risk, gait variability, motor control, neuromuscular function, nonlinear dynamics, plantar flexor force, sample entropy, network physiology

## Abstract

**Introduction:**

Falls are a leading cause of injury, disability, and loss of independence in older adults, and are closely linked to age-related declines in neuromuscular function. Underlying these declines is a loss of physiological complexity, reflected in more predictable and repetitive temporal structures (i.e., decreased entropy) of motor output. Within a network physiology framework, this loss of complexity may manifest as reduced network dimensionality. It may also be consistent with an increased co-variation between motor outputs in different tasks. Therefore, the present study examined the relationship between the complexity of plantar flexor torque and gait in young and older adults.

**Methods:**

Thirty-eight healthy older adults and forty-six healthy young adults participated in a single laboratory session. Plantar flexor torque and gait were assessed in separate tasks: submaximal isometric contractions and continuous treadmill walking, respectively. Complexity was inferred from sample entropy, which quantifies the temporal structure of both signals.

**Results:**

A positive Bayesian correlation between torque and gait complexity was observed in older adults (τ = 0.245, BF_10_ = 3.985). In contrast, an absence of such correlation was found in young adults (τ = −0.079, BF_10_ = 0.257). The Bayesian comparison of the two correlations revealed a stronger association between gait and torque in older adults compared to young adults (Δτ = 0.324, BF_10_ = 3.456).

**Discussion:**

These findings are consistent with, but do not directly demonstrate, an age-related loss of dimensionality in the neuromuscular network, in which motor outputs that vary independently in young adults co-vary in older adults. By identifying plantar-flexor torque complexity as a potential modifiable factor that is associated with gait complexity in older adults, these results motivate longitudinal work testing whether interventions that target torque control, such as resistance training, can elicit corresponding improvements in gait and thereby reduce fall risk.

## 1 Introduction

Aging leads to a loss of physiological complexity accompanied by impaired motor control (Lipsitz and Goldberger, 1992; Fiogbé et al., 2021). In non-linear dynamics, complexity is described by the structure of temporal sequences (Stergiou et al., 2004). A complex system is characterized through neither too unpredictable (i.e., irregular) nor too predictable (i.e., regular) temporal patterns (Stergiou et al., 2006). Healthy young adults are thought to present such complex patterns with an optimal amount of unpredictability allowing for the system’s capacity to flexibly adapt to physiological stress. On the contrary, aging is associated with a general decrease of physiological complexity (Lipsitz and Goldberger, 1992; Stergiou et al., 2006). That is especially evident in research assessing gait entropy, as with aging the number of biologic inputs decreases causing simplified and more predictable temporal structures with a rigid stepping pattern (Acharya et al., 2013). This may indicate an overly regular system with impaired capacity to adapt to possible environmental constraints (Davids et al., 2003). This loss of adaptability may not just indicate a risk of falls, but a decline in functional capacity with increased risk of frailty (Lipsitz, 2002).

Lower limb strength is an important factor to maintain functional capacity and prevent falls (Suzuki et al., 2001; Namayeshi et al., 2023). Specifically, the plantar flexor muscles are important for stabilization and forward propulsion in mid- and terminal stance phase (Francis et al., 2013). Therefore, plantar flexor strength has been shown to be positively correlated to walking speed in older adults (Bendall et al., 1989; Suzuki et al., 2001; Tavakkoli Oskouei et al., 2021). Furthermore, Namayeshi and colleagues (2023) demonstrated that reduced plantar flexor strength is associated with a limited capacity to generate sufficient ankle joint moments, which may compromise successful recovery following a trip during walking. Importantly, the authors suggested that not only the magnitude of force output affects gait, but specifically the temporal characteristics of the ankle response appear to play a critical role in fall prevention.

One method used to quantify temporal characteristics of a signal is sample entropy (SampEn), a nonlinear analysis technique that indicates the inverse probability that short sequences of data points within a signal will repeat themselves throughout its length (Richman and Moorman, 2000). A low SampEn value (towards 0) reflects a highly predictable signal, in which similar sequences of data points recur consistently, whereas a high SampEn value (towards infinity) reflects an unpredictable signal, in which sequences of data points rarely repeat. Because SampEn characterizes the temporal structure of a signal rather than its overall magnitude, it can reveal patterns that would remain undetected by traditional summary statistics such as the mean or coefficient of variation alone. For example, SampEn has been used to distinguish healthy from pathological cardiac function based on the regularity of the heartbeat interval signal, with patients with congestive heart failure showing lower entropy (i.e., a more regular, predictable beat-to-beat pattern) than individuals in normal sinus rhythm (Zhao et al., 2015).

Regarding the temporal characteristics of torque and gait signals with aging, research analyzing the pattern predictability (i.e., entropy) shows similar results for both signals. In addition to the previously mentioned change to more predictable gait patterns reflected by decreased entropy (Acharya et al., 2013), force fluctuations also change towards more predictable patterns showing decreased entropy (Fiogbé et al., 2021). This suggests that aging affects the complexity of gait and force not only in isolation but potentially reflects a broader reorganization of the neuromuscular system. Indeed, Garcia-Retortillo and colleagues (2025) found that aging leads to an overall decline in inter-muscular coordination and in an increased stratification of the muscular network suggesting less flexible and adaptable systems. On a global scale, the network physiology framework characterizes healthy function within physiological complexity as flexible, appropriately weighted interactions among physiological systems, subsystems or network components, whereas aging is suggested to compress this network toward stronger, more rigid coupling and reduced dimensionality (Balagué et al., 2020). In the present study, we do not aim to measure inter-system coupling directly: torque and gait are analyzed in separate tasks, so a direct, time-resolved coupling analysis (e.g., based on time-delay stability between simultaneously acquired signals) is not intended. Instead, we test a prediction that follows from the framework-that if the neuromuscular network loses dimensionality with age, the temporal complexity of motor output in distinct tasks should cease to vary independently and instead co-vary across individuals. A positive association between plantar flexor torque complexity and gait complexity in older adults, absent in young adults, would therefore not constitute direct evidence of subsystem coupling, but be consistent with reduced network dimensionality.

Understanding a potential co-variation of motor output across tasks with aging may support more holistic approaches to health interventions for older adults. Therefore, the present study assessed the between-subjects association between the complexity (inferred from sample entropy) of plantar flexor torque and gait dynamics in young and older adults. We hypothesized that plantar flexor torque complexity would be positively correlated to gait complexity in older adults, whereas no such correlation was expected in young adults. Accordingly, the strength of the association between torque and gait complexity was expected to differ between the two age groups.

## 2 Materials and methods

### 2.1 Participants

An a priori power analysis for correlations of two independent Pearson r’s was conducted in G*Power software (Version 3.1, Heinrich Heine University Düsseldorf, Düsseldorf, Germany), assuming a statistical power of at least 80% and an effect size (q) of 0.6 at a significance level of α = 0.05. This analysis indicated the need for at least thirty-eight participants per group. Therefore, thirty-eight healthy older (60–87 years) and forty-six healthy young adults (18–34 years) took part in this investigation.

Inclusion criteria were being aged between 18 and 35 years for the young adult group and at least 60 years for the older adult group. Exclusion criteria were severe cardiovascular or pulmonary disease, lower limb disabilities, neurological disorders, cognitive impairment and orthopedic concerns, which might limit force production. Condition for participation was a signed informed consent approved by the ethics committee of the Egas Moniz School of Health and Science (Date: 29 June 2023/No. 1236) and in conformity with the Declaration of Helsinki.

Participants were recruited from the community via word of mouth. Of 50 older adults screened, 12 were excluded based on the exclusion criteria, yielding 38 participants. Of 47 young adults screened, 1 was excluded due to the exclusion criteria, yielding 46 participants. No participants withdrew or were excluded after enrolment in either group, and all participants completed the study protocol.

### 2.2 Experimental design

Participants were instructed to avoid heavy exercise 48 h prior to testing, and drugs, caffeine, tea or other stimulating substances 24 h before the evaluation. Participation in the present study consisted of a single visit to the laboratory. Participants were familiarized with the tasks. In terms of treadmill familiarization, participants walked for 5 min to accommodate to the treadmill and following, the preferred walking speed was determined (Jordan et al., 2007; Vaz et al., 2020; Jordão et al., 2023). Briefly, treadmill speed was increased in 0.1 m/s increments until participants reported the speed as “too fast to be comfortable,” then decreased until participants judged “too slow to be comfortable.” This procedure was repeated three times, and the mean speed was used as the participant’s preferred walking speed. During the procedure, participants were blinded to speed changes. Subsequently, the force and gait assessments were carried out in a randomized order for all participants. The gait assessment consisted of 12 min of continuous walking at the preferred walking speed, from which gait complexity was extracted. The plantarflexion force assessment incorporated maximum voluntary isometric contractions (MVIC) and submaximal isometric contractions at 40% of MVIC in this order (Oliveira et al., 2024; Bauer et al., 2025). Torque complexity was extracted from the submaximal isometric contractions. Between gait and plantarflexion force assessments a 5-min rest period was carried out.

### 2.3 Data collection

To assess plantarflexion torque, a HUMAC NORM Isokinetic Dynamometer (CSMI, Stoughton, MA) was used. Setting and calibration were performed according to the manufacturer’s information for each participant. Individuals were seated in a reclined position with the hip and knee flexed at 60° (0° representing neutral hip position and full knee extension). The lateral malleolus was aligned with the lever arm rotation axis (Figure 1A). To maintain the position during contraction, participants were secured with straps around thigh, hip and shoulders. The dominant foot was strapped to the lever arm. The ankle 0° reference position was defined as the foot positioned perpendicular to the shank (anatomical neutral). All tests were carried out isometrically at 15° of dorsiflexion to support optimal conditions for force production (Sale et al., 1982; Landin et al., 2015).

**FIGURE 1:**
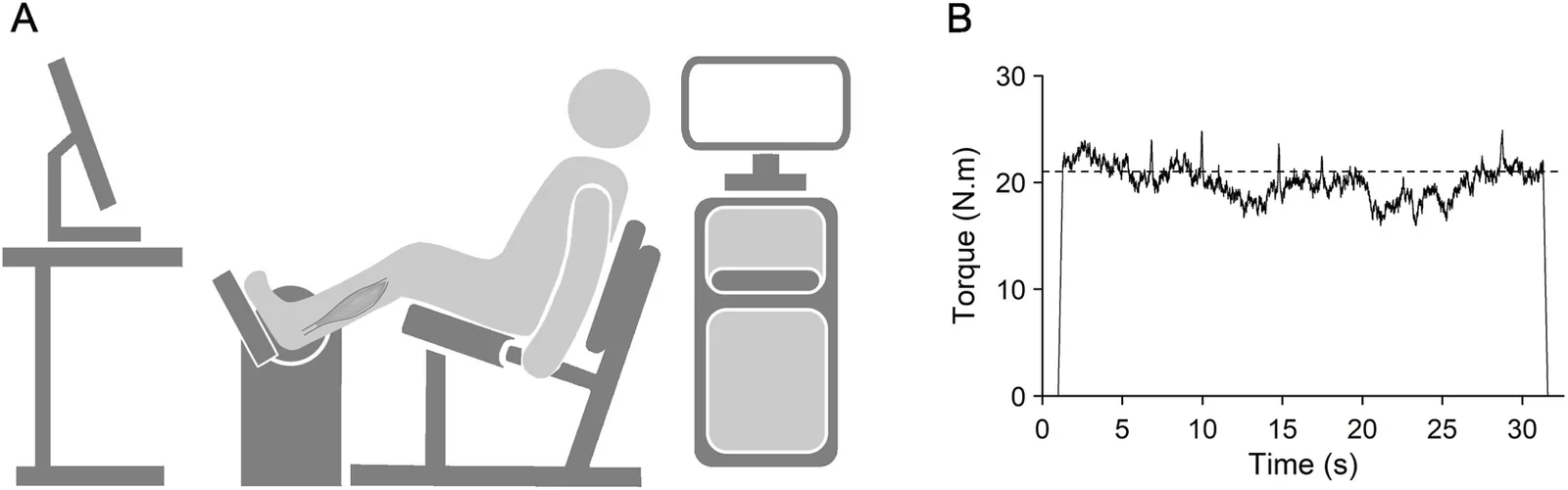
**(A)** Schematic representation of the participant seated on the isokinetic dynamometer. **(B)** Illustrative representation of torque-time curve in submaximal isometric contraction with the dotted line exemplifying individual intensity (i.e., 40% of maximum voluntary contraction).

All dynamometry trials consisted of plantarflexion contractions. MVICs were carried out with strong verbal encouragement. Participants carried out MVICs until the difference between repetitions was ≤5% (i.e., consistent). If this criterion was met within three repetitions, the highest torque value of the two consistent trials was used as reference. If this criterion was not met within three repetitions, the middle repetition was used as reference to determine the individual intensity (i.e., 40% of MVIC) for the submaximal contractions. The submaximal isometric contractions were push plantarflexion tasks, in which the dynamometer’s lever arm was electronically locked at the set angle and participants generated force by pushing against it in the plantarflexion direction, without any joint movement. The torque output was plotted on a screen, and participants were asked to comply with the individual intensity, which appeared as a line on the screen, for 30 s (Figure 1B). Two trials were carried out. Rest between all trials was defined as 1 min. All signals were sampled at 1 kHz with CED Micro 1401 data acquisition system (Cambridge Electronic Design, Cambridge, United Kingdom). Data was collected with Spike2 software (Version 7; Cambridge Electronic Design, Cambridge, United Kingdom).

The gait task was conducted on an instrumented treadmill (Bertec Corporation, Columbus, OH). Kinetic data was collected in Vicon Nexus (Version 2.14; Vicon Motion Systems, Oxford, United Kingdom) and sampled at 1 kHz.

### 2.4 Data analysis

Data analysis of all signals was conducted with MATLAB R2023a programming software (MathWorks, Natick, MA). No filters were applied and the raw data was used for further processing. Concerning torque data of the submaximal isometric contractions, the 30 s contraction period analyzed corresponds to the sustained portion of the recorded torque signal, excluding the initial transient associated with reaching and settling at the target torque and the final relaxation phase, which were cropped prior to analysis. Because MATLAB indexing includes both the start and end samples, cropping produced 30001 samples (1,000 Hz x ∼30 s) rather than 30000 samples (1,000 Hz × 30 s). Following, a power spectral analysis was performed on the cropped torque signal to determine the sampling frequency. Briefly, using an FFT-based periodogram, the cumulative power spectrum was computed, and the cutoff frequency was defined as the frequency below which 99.9% of the cumulative spectral power was contained. The analysis revealed a maximal frequency across all participants and trials of 3.45 Hz. Following the recommendation of Stergiou et al. (2004) of a sampling frequency approximately five times greater than the maximal frequency, data was down sampled to 25 Hz. After down sampling the torque time series consisted of 751 data points (25 Hz x ∼30 s). The first and last points were removed to minimize potential edge artifacts, yielding 749 data points for analysis. Torque complexity was derived from the temporal structure of the cropped and down sampled torque time series. This structure was analyzed using sample entropy (SampEn), which indicates the inverse probability that short sequences of data points are repeated throughout the signal length (Richman and Moorman, 2000). Lower SampEn (towards 0) is the consequence of similar distances between repetitions of those sequences, which indicates a more predictable pattern of torque output. A higher SampEn value (towards infinity) results from large differences of distances between repetitions of sequences, which designates a more unpredictable pattern of torque output. For the determination of SampEn, in all torque time series of the present study, the values were set as follows: pattern length (m) at 2, error tolerance (r) at 0.2 x standard deviation (SD) and data length (N) at 749 data points (i.e., 25 Hz x ∼30 s) (Raffalt et al., 2023). The mean of the SampEn values from the two submaximal trials was used for statistical analysis.

Regarding gait analysis, inter-stride intervals (ISIs) were calculated as the time between consecutive heel strikes of the same foot. Heel strikes were identified from the vertical ground reaction force signal recorded via a force plate under the dominant leg as instances where the signal fell within a threshold band of 8–12 N and was increasing, with toe offs identified using the same threshold band and a decreasing slope criterion. This band and slope approach was used to distinguish heel strikes from toe offs and to reduce spurious detections near a fixed threshold. Automatically detected events were visually verified against the raw force signal for each participant. ISI time series ranged from 558 to 820 data points depending on the PWS. All ISI time series were cut to 550 data points to ensure a consistent time series length across all trials for further data processing. As with torque complexity, gait complexity was inferred from SampEn. The following input parameters were set for processing all gait time series of the present study: pattern length (m) at 2, error tolerance (r) at 0.3 x SD and data length (N) at 550 points (Yentes et al., 2013). Different *r* values were used for torque (0.2) and gait (0.3) time series because *r* is conventionally scaled to the properties of each signal, and an appropriate value depends on the noise characteristics, temporal dynamics and sampling protocol of the specific measure (Yentes et al., 2013; Raffalt et al., 2023).

### 2.5 Statistical analysis

All statistical analyses were performed using Jamovi (Version 2.6; The jamovi project, 2024). Descriptive statistics, including means and standard deviations, were calculated to summarize the data. Differences in demographic and clinical characteristics between age groups were tested with the Bayesian independent samples t-test or whenever normality was violated, with the non-parametric equivalent (i.e., Bayesian Mann-Whitney U test). For the nominal variable sex, a Bayesian contingency table test (independent multinomial sampling, uniform prior) was used. Bayesian statistics were used because one of our hypotheses predicted the absence of a correlation, which cannot be evaluated with frequentist null hypothesis significance testing, whereas Bayes factors allow direct quantification of relative evidence for either hypothesis, including the null hypothesis (Wagenmakers et al., 2011). Data were tested for normality using the Shapiro-Wilk test. As this assumption was violated for gait (W = 0.893, p < 0.001) and torque SampEn (W = 0.935, p = 0.013) in young adults and for gait SampEn (W = 0.929, p = 0.018) in older adults, Kendall’s tau-b was used for Bayesian correlations (van Doorn et al., 2018). To compare the strength of association between age groups, the posterior distributions of the two correlations were used to derive a posterior distribution of their difference, from which a Bayes factor was computed. For interpretation of the statistics BF_10_ was used, where values greater than 1 indicate support for the alternative and values lower than 1 for the null hypothesis. BF_10_ values equal or greater than 3 and 10 are considered moderate and strong evidence for the alternative hypothesis, respectively. BF_10_ values below 0.33 and 0.10 are considered moderate and strong evidence for the null hypothesis, respectively (Lee and Wagenmakers, 2014). Two-tailed Bayesian tests were used for hypotheses predicting absence of a difference, whereas one-tailed tests were used for prespecified directional hypotheses. The directional confirmatory hypothesis was that plantar-flexor torque complexity and gait complexity would covary positively, based on strong evidence from the network physiology framework and prior work on age-related reductions in physiological complexity and adaptive capacity (Lipsitz and Goldberger, 1992; Lipsitz, 2002; Rutenberg et al., 2018; Balagué et al., 2020). A negative association would indicate that higher torque complexity is associated with lower gait complexity, which would be interpreted as evidence against shared positive covariation of complexity across tasks and would lead to the same conclusion as a null association. Therefore, the unpredicted tail was considered redundant for the present confirmatory hypothesis and one-tailed tests were used, consistent with recommendations for directional tests when effects in the opposite direction would not support a distinct theoretical conclusion (Kimmel, 1957; Ruxton and Neuhäuser, 2010). Accordingly, for directional hypotheses (one-tailed), the prior was defined on the interval [0, 1] and for non-directional hypotheses (two-tailed), the prior was defined on the interval [−1, 1] (van Doorn et al., 2018).

## 3 Results

### 3.1 Demographic characteristics

The demographic and clinical characteristics of the older and young adult groups are presented in Table 1. Variables include age, sex, height, weight, BMI, MVIC, and PWS. BF_10_ values indicated strong-to-decisive evidence for group differences in age, height, BMI, MVIC, and PWS, and evidence favoring the null for sex and weight.

**TABLE 1:** Demographic and clinical characteristics. Data are presented as mean ± SD, except for sex, which is presented as % of females per group. δ indicates the posterior median of the standardized effect size (with 95% credible interval). BMI, body mass index; MVIC, maximal voluntary isometric contraction; PWS, preferred walking speed.

Variable	Older adults (n = 38)	Young adults (n = 46)	δ [median, 95% CI]	BF_10_
Age (years)	70.4 ± 5.9	26.0 ± 4.7	+8.1 [7.1, 9.2]	>100
Sex (% female)	57%	50%	-	0.33
Height (m)	1.64 ± 0.08	1.71 ± 0.08	−0.74 [−1.26, −0.23]	16
Weight (kg)	71.7 ± 13.1	68.7 ± 12.0	+0.2 [−0.3, 0.7]	0.37
BMI (kg/m^2^)	26.6 ± 3.6	23.4 ± 2.6	+0.9 [0.3, 1.4]	58
MVIC (N·m)	52.9 ± 26.2	127.2 ± 38.0	−2.1 [−2.7, −1.3]	>100
PWS (km/h)	3.7 ± 0.8	4.4 ± 0.5	−1.1 [−1.6, −0.6]	>100

### 3.2 Correlations

For older adults, the Bayesian Kendall’s tau correlation revealed moderate evidence for a positive association between gait SampEn and torque SampEn (τ = 0.245, BF_10_ = 3.985, 95% CI (0.042, 0.435), one-tailed). For young adults, the Bayesian Kendall’s tau correlation showed moderate evidence for the absence of a correlation between gait SampEn and torque SampEn (τ = −0.079, BF_10_ = 0.257, 95% CI (-0.267, 0.114)). The correlation results are illustrated in Figure 2. The Bayesian comparison of the two correlations revealed moderate evidence that the association between gait SampEn and torque SampEn was stronger in older than young adults (Δτ = 0.324, BF_10_ = 3.456, 95% CI [0.074, 0.624], one-tailed).

**FIGURE 2:**
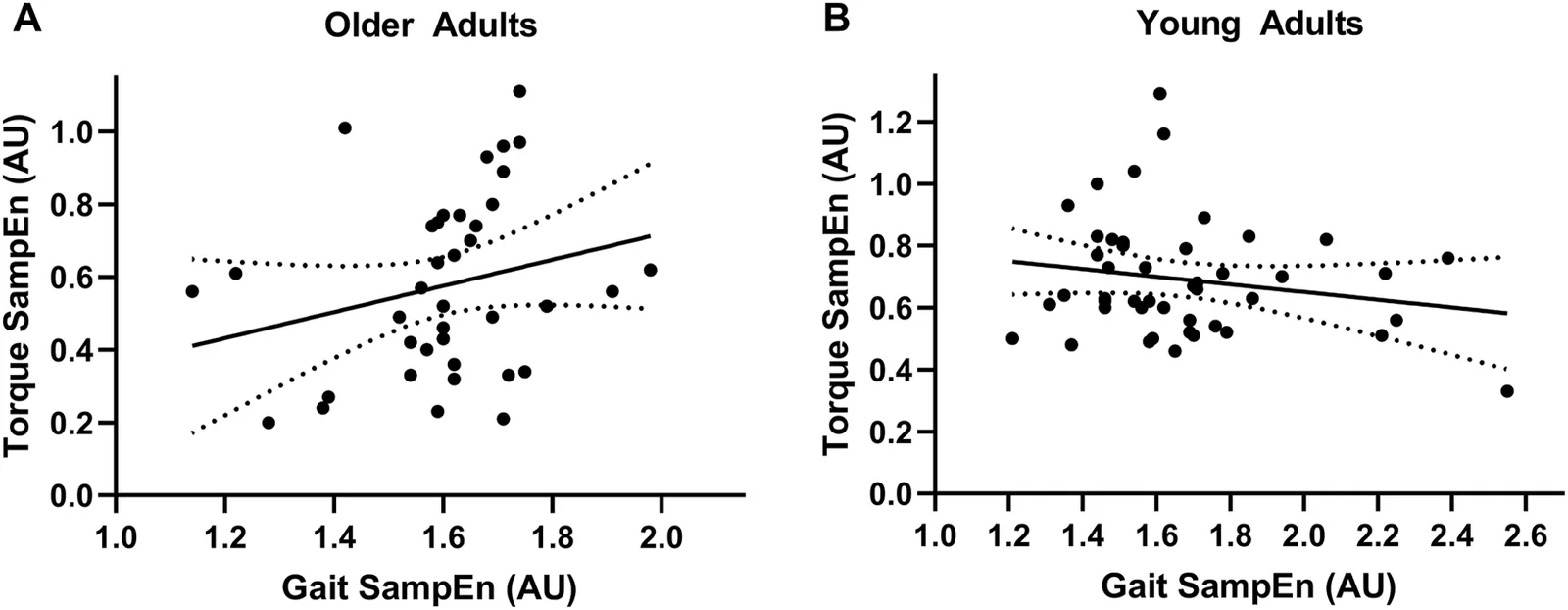
Scatter plots illustrating the relationship between torque and gait Sample Entropy (SampEn) for **(A)** older adults (τ = 0.245, BF_10_ = 3.985) and **(B)** young adults (τ = −0.079, BF_10_ = 0.257). Each data point represents an individual participant. The solid line represents the ordinary least-squares linear regression trend, shown for visualization purposes only and independent of the Bayesian Kendall’s tau correlation reported above, with 95% confidence interval (dotted lines) of the regression line.

## 4 Discussion

The present study assessed the relation between the complexity (inferred from Sample Entropy) of plantarflexion torque and gait, in young and older adults. We hypothesized that plantar flexor torque complexity is positively correlated to gait complexity in older adults. This hypothesis was supported, as the present results show moderate evidence for a positive association (BF_10_ = 3.985) between torque and gait complexity in the group of older adults (Figure 2A). Furthermore, we hypothesized no correlation between torque and gait complexity in young adults. This hypothesis was also supported, as we found moderate evidence for the absence of such correlation (BF_10_ = 0.257) in the group of young adults (Figure 2B).

Aging is associated with an overly predictive and regular (i.e., decreased entropy) torque and gait patterns (Acharya et al., 2013; Fiogbé et al., 2021), which is consistent with a decrease in complexity in various domains and subsystems (Lipsitz and Goldberger, 1992; Lipsitz, 2002). Integrating this information at the level of the whole organism, network physiology suggests that a less complex, overly predictable system reflects stronger, more rigid interactions between network components with reduced dimensionality (Balagué et al., 2020). The present between-subjects association between torque and gait complexity in older adults is consistent with this prediction: motor outputs that vary independently in young adults appear to co-vary across older individuals. This pattern is consistent with reduced network dimensionality, rather than direct evidence of within-person dynamic coupling between the force-producing and gait-controlling subsystems, which our study design cannot address. Rutenberg and colleagues’ (2018) network model of health provides a complementary theoretical perspective: dysfunction in one domain is proposed to propagate across connected domains, with highly connected domains linked to the risk of mortality. Our finding that torque and gait complexity begin to co-vary in aging is consistent with the general prediction that neuromuscular subsystems become less independent with age, though establishing propagation of dysfunction would require longitudinal data.

In the present study, the different tasks involving the neuromuscular system were a plantar-flexor force and a gait task. Aging is associated with a loss of plantar-flexor strength (Vandervoort, 2002) and gait speed (Bohannon, 1997), consistent with the present study’s strong evidence for decreased plantar-flexion MVIC and PWS in the older adult cohort, when compared to young adults. These changes are considered age-related and reflect the physiological remodeling of the neuromuscular system that occurs with advancing age. To ensure comparability between groups, submaximal contractions, from which torque complexity was extracted, were calculated as a percentage of each participant’s MVIC, and PWS was determined individually for each participant. Although older adults walked slower than young adults, time series were cut to the same number of data points to ensure the validity of the sample entropy algorithm.

The Bayesian comparison of the two correlations revealed moderate evidence for a stronger association (BF_10_ = 3.456) between gait and torque complexity in older adults compared to young adults. In young adults, the complexity of motor output in a force task appears to vary independently from that of a gait task, suggesting task-specific control that may depend on motor skill and the behavioral repertoire available for each task (Stergiou et al., 2006). On the contrary, in older adults, low torque complexity tended to co-occur with low gait complexity across individuals, and vice versa. This population-level co-variation across two separately analyzed motor tasks is consistent with a reduction in the number of effective dimensions of the underlying neuromuscular network (Balagué et al., 2020), so that outputs previously driven by task-specific control become increasingly constrained by shared, lower-dimensional factors. This aligns with the findings of Garcia-Retortillo and colleagues (2025) that aging leads to an overall decline in inter-muscular coordination and in an increased stratification of the muscular network. Further possible mechanisms for such a reduction in effective dimensionality could lie within motor unit discharge characteristics, as force steadiness is thought to be influenced by the variability and the common modulation of discharge (Enoka et al., 2003). Furthermore, a loss of physiological complexity with aging is suggested to be linked with a decreased quantity of neurons and altered synaptic characteristics (Lipsitz and Goldberger, 1992). These age-related physiological changes might be related to an effective decrease in degrees of freedom, which remains to be evaluated in future studies.

It is important to distinguish the strength of evidence for a correlation from the magnitude of that correlation. The Bayes factor (BF_10_ = 3.985) indicates moderate evidence favoring the presence of an association between torque and gait complexity in older adults, but does not indicate whether the association is strong. The point estimate (τ = 0.245) reflects a relatively modest effect, and the 95% credible interval [0.042, 0.435] is wide and extends close to zero, indicating considerable uncertainty around the true magnitude of the association. Similarly, the Bayesian correlation comparison shows a wide 95% credible interval [0.074, 0.624]. Therefore, while the present findings support the existence of a link between torque and gait complexity in older adults and a stronger association between torque and gait complexity in older adults compared to young adults, the clinical significance of this association remains to be established in larger samples. Moreover, several sample characteristics may account for this limited magnitude. The older adults analyzed in the present study fell at the lower end of the older adult age range with a mean age of 70.4 years. Although, the Bayesian t-test on BMI showed moderate evidence for a difference between young and older adults, the mean BMI of OAs (26.6 kg/m^2^) is within the suggestion for a healthy BMI of older adults (Porter Starr and Bales, 2015) considered to avoid risk of functional impairments, gait problems and a decrease of muscle strength (Kıskaç et al., 2022). Furthermore, group differences in BMI were largely driven by height differences, as weight showed anecdotal evidence for no group differences. Considering the low mean age, the healthy mean BMI and the inclusion of only healthy older adults in the present study, we might speculate that the older adult cohort had not yet undergone the more pronounced neuromuscular changes characteristic of advanced or clinically compromised aging, which may account for the limited strength of the observed association. On the contrary, other third factors, which were not assessed in the present study, such as physical activity level, cognitive function, general health status or shared methodological factors between torque and gait, cannot be ruled out as additional contributors to the observed co-variation.

The a priori sample size calculation was based on the comparison of the two independent group correlations (q = 0.6). In the present study, this comparison was carried out as Bayesian correlation comparison (Δτ = 0.324, BF_10_ = 3.456). The observed effect corresponded to a somewhat smaller q (≈0.52) than assumed, yielding an estimated achieved power of approximately 74% rather than the targeted 80%. This modest shortfall in power is consistent with the moderate, rather than strong, level of evidence obtained for the between-group difference and suggests some caution in interpreting its precision, though the design remained reasonably well-suited to the comparison it was intended to test. Future studies with larger samples would help improve the precision and robustness of the estimate. Furthermore, the analyzed population in the present study consisted of healthy young and healthy older adults. The between-subjects co-variation of motor outputs may be more pronounced in clinical populations, where reductions in network dimensionality are expected to be more advanced, possibly indicating a greater need to evaluate these populations. Moreover, gait was carried out in laboratory conditions, on an instrumented treadmill. Despite the implementation of an adequate familiarization protocol, the extent to which these findings generalize to overground walking under real-life conditions remains uncertain and warrants dedicated investigation. It should be noted that, based on the characteristics of the system and the type of constraints, an increase or a decrease of complexity can occur (Vaillancourt and Newell, 2002) and therefore, a loss of complexity is not intrinsically pathological. Therefore, caution is warranted in the interpretation of data from different populations and from tasks with different characteristics. In addition to these future directions, the present design does not address within-person dynamic coupling between the force-producing and gait-controlling subsystems. As a next step, future studies should acquire simultaneous force and gait signals combined with time-resolved coupling analyses (e.g., time-delay stability) to assess the over-synchronization hypothesis in a more direct way. Most importantly, the cross-sectional nature of the present design leaves open whether torque complexity causally contributes to gait complexity or whether both reflect shared upstream neuromuscular factors. Future work should therefore adopt interventional designs in which torque control is deliberately modulated and concurrent changes in gait complexity are examined. Resistance training may represent a promising avenue to explore in this context.

The present study follows the call from Balagué and colleagues (2020) to shift from isolated physiological measures towards an integrated network approach. The correlation between torque and gait complexity in older adults, and its absence in young adults, is consistent with a loss of dimensionality in the neuromuscular network with age. While aging itself cannot be reversed, its neuromuscular manifestations may be modifiable to mitigate potential limiting factors. By identifying plantar-flexor torque complexity as a potentially modifiable factor that is associated with gait complexity, the present findings motivate the targeted evaluation of interventions, such as resistance training, designed to restore complexity in motor output and, in turn, potentially improve gait and reduce fall risk in older adults.

## Data Availability

The raw data supporting the conclusions of this article will be made available by the authors, upon a reasonable request. Requests to access the datasets should be directed to jvaz@egasmoniz.edu.pt.

## Supplementary material

SUPPLEMENTARY FIGURE S1 Illustrative representation of an inter-stride interval time series during treadmill walking at preferred walking speed.
